# Prolapse of fallopian tube through abdominal wound after caesarean section mimicking scar endometriosis: a case report

**DOI:** 10.1186/s13256-015-0769-3

**Published:** 2015-12-17

**Authors:** Lajya Devi Goyal, Shalini Maheshwari, Sharanjit Kaur, Harpreet Kaur

**Affiliations:** 1 Department of Obstetrics and Gynaecology, Gurugobind Singh Médical Hospital, Faridkot, Punjab 151203 India; 2 University Collège of Nursing, Faridkot, Punjab 151203 India

**Keywords:** Fallopian tube, Prolapse, Scar endometriosis

## Abstract

**Introduction:**

Prolapse of the fallopian tube after hysterectomy is a rare but known complication. Cases of prolapse of the fallopian tube through the vaginal vault have been reported after abdominal, vaginal or laparoscopic hysterectomies. This is the first case report to the best of our knowledge on the prolapse of a fallopian tube through an abdominal wound after caesarean section.

**Case presentation:**

We report a case of the prolapse of the fimbrial end of a fallopian tube through an abdominal scar after caesarean section mimicking scar endometriosis. A 24-year-old primipara South Asian woman of Punjabi ethnicity presented to our institute with a fleshy mass protruding through her abdominal scar and bleeding from the mass during menstruation for the past 5 months. She underwent a caesarean section 6 months earlier for breech presentation. Her history revealed she had wound dehiscence on the sixth postoperative day. The major portion of her wound healed in 1 month leaving a 2 cm area in the middle of her vertical scar. An abdominal examination revealed a 2×2 cm fleshy mass protruding through the middle part of her infraumbilical abdominal scar. At the time of the surgery we found that the fimbrial end of her left fallopian tube was protruding through her abdominal scar.

**Conclusion:**

Awareness of this complication may prevent improper management of wound dehiscence and such complication causing prolonged agony to the patient.

## Introduction

Prolapse of the fallopian tube after hysterectomy is a rare but known complication. Cases of prolapse of the fallopian tube through the vaginal vault have been reported after abdominal, vaginal or laparoscopic hysterectomies [1–3]. We report a case of prolapsed fimbrial end of the fallopian tube through an abdominal scar after caesarean section which mimicked scar endometriosis and is very unusual. This is the first case report to the best of our knowledge on the prolapse of a fallopian tube through an abdominal wound after caesarean section.

## Case presentation

**Fig. 1:**
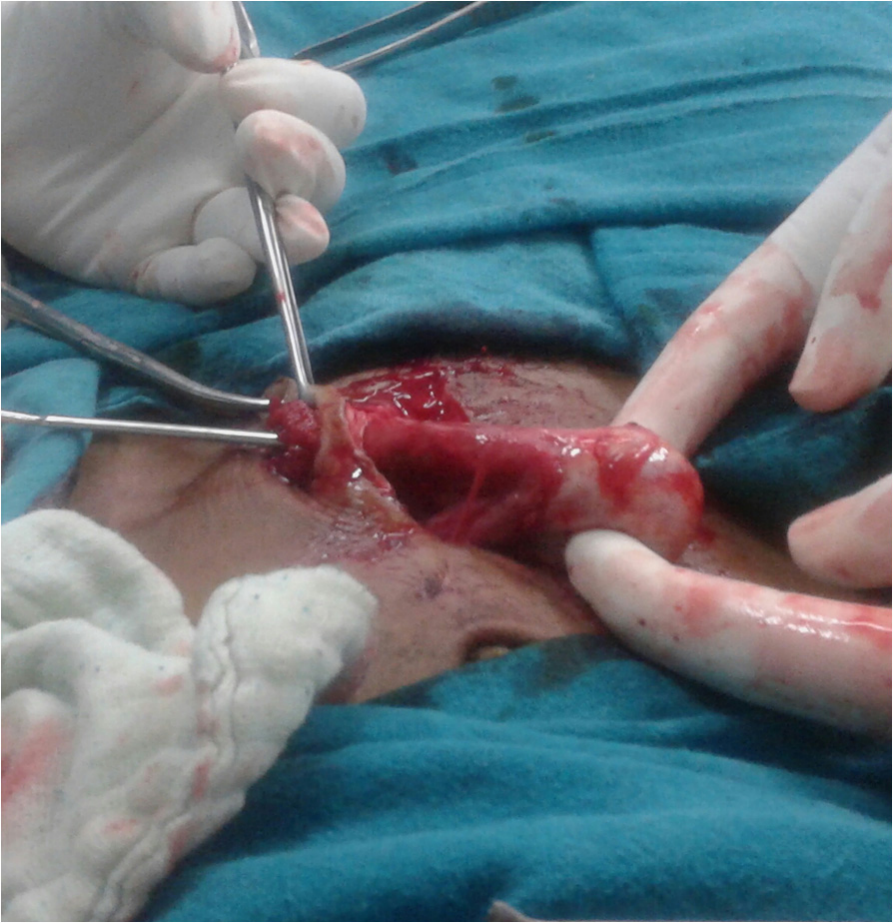
Intraoperative photograph showing prolapsed fallopian tube through abdominal wound

A 24-year-old South Asian woman of Punjabi ethnicity presented with fleshy mass protruding through midline vertical abdominal scar and bleeding from the mass during menstruation for the past 5 months. She was primigravida; she underwent a caesarean section 6 months earlier at term gestation for breech presentation in a local hospital. She delivered a normal healthy baby boy and the immediate postpartum period was uneventful. On the sixth postoperative day she noticed serosanguinous discharge from her abdominal wound and wound dehiscence was diagnosed. She was managed conservatively and the wound was left for secondary healing by the attending physician. Her history revealed that she was given antibiotic coverage during this time. The major portion of her wound healed in 1 month leaving a 2 cm area in the middle of her vertical scar. An investigation at the time of her caesarean section revealed that she was anemic (hemoglobin 8 gm %). Peripheral blood film revealed microcytic hypochromic anemia. On admission to our institute she was emaciated, thin built, anemic and her vital signs were normal. An abdominal examination revealed a 2×2 cm fleshy mass protruding through the middle part of her infraumbilical abdominal scar. On per vaginal examination her uterus seemed attached to the anterior abdominal wall at the scar site and with cervical movement the mass was getting retracted into her abdomen. With her history of menstruation through the abdominal wound a provisional diagnosis of scar endometriosis/uterocutaneous fistula was made and ultrasonography (USG) and fistulogram were suggested. USG showed normal uterus and adnexa and fistulogram showed communication with intraperitoneal cavity. A tissue biopsy revealed granuloma. She was planned for excision of the fistula tract and repair. On an operating table methylene blue dye was injected through the wound to mark the fistulous tract and dye was found to be escaping through her vagina confirming communication with uterine cavity. An elliptical incision was made around the fleshy mass and on entering the abdominal cavity, the left side of her uterus was adherent with her anterior abdominal wall at the scar site and the fimbrial end of her left fallopian tube was found to be protruding through the abdominal scar. A probe (dilator) was passed through the tube and diagnosis was confirmed (Fig. 1). The tube was pulled inside and adhesiolysis of uterine adhesions from anterior abdominal wall was done. The scar edges were freshened and the incision was closed in layers. She had an uneventful recovery. She was followed up monthly for 3 months and had no complaints.

## Discussion

Pozzi in 1902 described the first report of postoperative prolapse of fallopian tube after vaginal hysterectomy [4]. Since then, there have been approximately 100 case reports of prolapse of fallopian tube after hysterectomy and the prevalence after hysterectomy is reported to be approximately 0.3 % [5]. There is no literature to the best of our knowledge on prolapsed fimbrial end of the fallopian tube through caesarean section wound. There could be multiple predisposing factors that lead to fallopian tube prolapse. Ouldamer *et al*. [6] did a systemic review on prolapse of fallopian tube after hysterectomy and suggested multiple contributing factors including defective operative technique, poor nutritional status, pelvic infection, wound hematoma, uncontrolled diabetes, chronic cough and constipation. The poor physical status of the patient, anemia malnutrition, postoperative wound infection and letting the wound heal by secondary intention could have been the contributing factors in this case. Diagnosis at the time of postoperative wound disruption was probably missed. In this case the symptoms mimicked scar endometriosis because it typically presented after caesarean section and there was menstrual bleeding through the wound. The differential diagnosis in such cases includes scar endometriosis, uterocutaneous fistula and wound granuloma. We have reported six cases of scar endometriosis with similar findings [7]; however, fallopian tube tissue is firmer than granulomatous and endometriotic tissue. The easy passage of a probe into the lumen of the fallopian tube may aid in establishing the diagnosis as in our case. Management depends on the presence of infected tissue on the exposed end. Partial or total salpingectomy may be required. In our index case, the fallopian tube was healthy so we cleaned the fimbrial end thoroughly and replaced it in the abdominal cavity.

## Conclusions

Prevention of tubal prolapse can be achieved by improving the general condition of the patient and treating anemia and infections during the pregnancy, using a proper technique of wound closure at the time of primary surgery and careful inspection in the case of wound dehiscence for burst abdomen. Awareness of this complication may prevent inadequate treatment of wound dehiscence and such complication causing agony in the patient’s life.

## Consent

Written informed consent was obtained from our patient for the publication of this case report and any accompanying images. A copy of the written consent is available for review by the Editor-in-Chief of this journal.
